# Enantioselective Labeling of Zebrafish for D-Phenylalanine Based on Graphene-Based Nanoplatform

**DOI:** 10.3390/molecules28093700

**Published:** 2023-04-25

**Authors:** Yuqing He, Ziqi Ye, Fei Zhu, Tianxiang Qiu, Xiyan Dai, Yue Xie, Shibiao Zou, Qingjian Dong, Weiying Zhang, Junkai Ma, Xiaowei Mao

**Affiliations:** 1 Hubei Key Laboratory of Environmental and Health Effects of Persistent Toxic Substances, School of Environment and Health, Jianghan University, Wuhan 430056, China; 2 Key Laboratory of Optoelectronic Chemical Materials and Devices of Ministry of Education, School of Life Science, Jianghan University, Wuhan 430056, China; 3 Hubei Key Laboratory of Embryonic Stem Cell Research, School of Basic Medical Science, Hubei University of Medicine, Shiyan 442000, China; 4 Department of Nuclear Medicine, Tongji Hospital, Tongji Medical College, Huazhong University of Science and Technology, Wuhan 430030, China; 5 Hubei Key Laboratory of Wudang Local Chinese Medicine Research, Department of Chemistry, School of Pharmacy, Hubei University of Medicine, Shiyan 442000, China

**Keywords:** chiral recognition, fluorescence imaging, γ-cyclodextrin, graphene oxide, amino acid enantiomers

## Abstract

Enantioselective labeling of important bioactive molecules in complex biological environments by artificial receptors has drawn great interest. From both the slight difference of enantiomers’ physicochemical properties and inherently complexity in living organism point of view, it is still a contemporary challenge for preparing practical chiral device that could be employed in the model animal due to diverse biological interference. Herein, we introduce γ-cyclodextrin onto graphene oxide for fabricating γ-cyclodextrin and graphene oxide assemblies, which provided an efficient nanoplatform for chiral labelling of D-phenylalanine with higher chiral discrimination ratio of K_D_/K_L_ = 8.21. Significantly, the chiral fluorescence quenching effect of this γ-CD-GO nanoplatform for D-phenylalanine enantiomer in zebrafish was 7.0-fold higher than L-isomer, which exhibiting real promise for producing practical enantio-differentiating graphene-based systems in a complex biological sample.

## 1. Introduction

The topic of chiral sources and its recognition mechanism in biological organism has drawn a great interest [1,2,3,4]. Amino acids (AAs) and their derivatives, served as important chiral bioactive substances with two non-superposable mirror-image forms, closely related to multiple metabolism and physiological functions in living organisms [5,6,7,8]. The different enantiomers of one AAs may play different roles in living system. For instance, L-amino acids are the vital building blocks of proteins, while free D-amino acids are considered as biomarkers for diseases [9,10,11]. To this end, numerous artificial chiral devices, exemplified as electroanalytical and colorimetric sensors, were developed to exhibit different responses toward chiral AAs enantiomers in an aqueous solution [12,13,14,15,16,17]. Among them, fluorescence imaging helped to visualize precise information and location and function of target molecules with high sensitivity, which had been most widely used in living organisms [18,19,20]. However, from both the similarity of enantiomers’ physicochemical properties and inherently complexity in living organism point of view, these chiral fluorescence imaging systems are still far from reaching the demands of chiral labeling of model animals with diverse interference [21,22,23]. Therefore, the development of an improved artificial receptor simultaneously possessing excellent analysis capacity of the AAs enantiomeric purity and strong anti-interference ability in living organism is of practical importance.

Graphene oxides (GO), composed of monolayers of carbon atoms, have drawn considerable research attention owing to its remarkable energy conversion property, electrochemically activity, and storage devices [24,25,26,27]. More important, GO also exhibits the excellent fluorescence quenching ability and transmembrane ability, which endowing it to be potentially substrate for constructing sensitive fluorescent sensing and bio-sensing nanoplatform [24,25,26,27]. For instance, Qu and co-workers developed amylose-functionalized graphene biosensor with highly optical sensitive sensing capability of L-tryptophan in water [28]. Immediately after that, Li et al. reported a functionated-GO nanodevice exhibited a strong chiral discrimination of D-Phe both in solution and in living cells [29]. Nevertheless, owning to the numerous key chiral recognition process needed to trace are performed in living system, enantioselective labeling in living organism performance of follow-on chiro-selective graphene-based probe is particularly important for an idealized nano-platform. Whereas selective imaging of chiral AAs enantiomers in living organisms is still open-wide.

Motivated by this task, we developed a convenient and effective procedure for assembling chiral GO-based nanodevice, which possesses efficient enantioselective fluorescence labelling ability for zebrafish. In our strategy, γ-cyclodextrin (γ-CD), with its intrinsic chirality and unique host-guest recognition ability [30,31,32,33], was used as enantioselective sensing functional groups for embellishing onto the surface of GO. Phenylalanine (Phe), closely related with hyperphenylalaninemia disease, is widely used as food or a feed additive in infusion fluids or for chemical synthesis of pharmaceutically active compounds, served as representative chiral substrates in our manuscript. Simultaneously, 1-dimethylamino-naphthalene-5-sulphonyl chloride, regard as fluorescent derivative group [32,33,34,35,36,37,38,39,40,41], was linked to AAs to produce fluorescent dansyl chloride-Phe. In this γ-cyclodextrin modified graphene oxide sensing system, γ-cyclodextrin recognition chiral AAs molecular providing stereoselectivity while graphene oxide translated it to optical fluorescent response. Moreover, the sensing ability of this nano-platform in zebrafish model animal is also attempted.

## 2. Results and Discussion

**Scheme 1:**
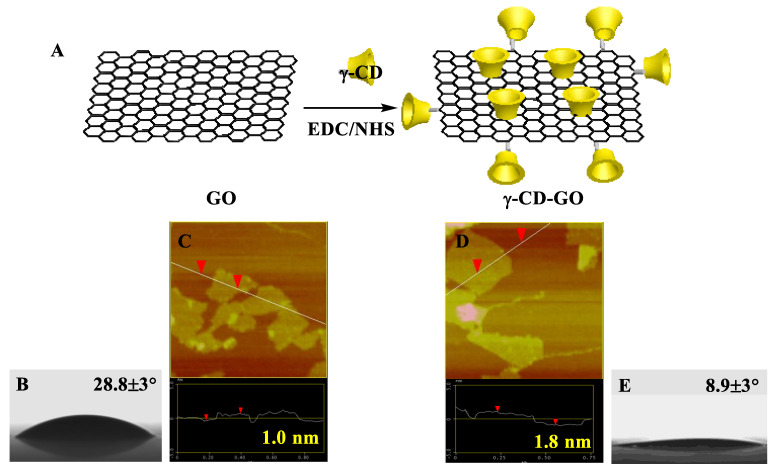
(**A**) Schematic synthesis route of the γ-CD-GO assemblies; The CA assay of (**B**) GO and (**E**) γ-CD–GO; AFM images of (**C**) GO, and (**D**) γ-CD-GO, respectively. The variation of AFM and CA characterization clearly demonstrates successfully chemical modification.

Considering the excellent size complementarity capability of γ-CD for phenylalanine chiral recognition [42], D/L-phenylalanine was selected as guest analytes in this manuscript. We synthesized fluorescent D/L-phenylalanine (F-D/L-Phe) derivatives by means of a typically dansyl chloride derivatization [32,33,34,35,36,37,38,39,40,41]. A bright yellow fluorescence was observed with the F-D/L-Phe solution, which stayed stable for more than three months with a good photostability [43,44]. The fabrication process of γ-CD modified graphene oxide (γ-CD-GO) chiral-recognition framework was depicted in Scheme 1. GO decorated with carboxyl groups was manufactured according to the previous literature [45,46,47,48], γ-CD molecules were subsequently introduced onto GO by the condensation reaction of amino and carboxyl reaction. The transmission electron microscopy (TEM) in Figure S1 clearly demonstrated the lamellar structure for GO. Furthermore, as atomic force microscopy (AFM) images (Scheme 1C,D) shown, the single-layer feature of GO was verified. Additionally, the height of GO was proved to be 1.0 ± 0.2 nm, which was in accordance with monolayer of GO [45,46,47,48]. However, the thickness of γ-CD-GO was clearly increased to 1.8 ± 0.2 nm after the chemical modification. Considering the thickness growth of γ-CD-GO and 0.79 nm for the height of γ-CD molecular [45,46,47,48], it is reasonably concluded that the thickness increase in γ-CD-GO was ascribed to the chemical derivatization by the γ-CD onto GO. Moreover, the contact angle (CA) characterization also validated the chemical derivatization process (Scheme 1B,E). The CA of GO is proved to be 28.8 ± 3°, while a remarkably CA decrease to 8.9 ± 3° after chemical functionalization, manifesting the γ-CD-GO is more hydrophilic due to the chemical decoration of hydrophilic γ-CD molecules [45,46,47,48]. Chemical functionalization procedure was also fully investigated by means of fourier infrared spectrum (FT-IR). Seen from the FT-IR analysis in Figure S2A, obvious conversion was demonstrated: the distinct peaks for native γ-CD were also found in γ-CD-GO around 1050 to 1346 cm^−1^, related to the C-O-C bond asymmetric stretching vibration [49,50]. More important, the stretching vibration of -COOH in GO at 1729 cm^−1^ was redshifted to 1680 cm^−1^ in γ-CD-GO, attributed to a dehydration reaction between carboxyl groups and amine groups to generate a covalent bond of amide. Raman spectrum data was also proved to have a GO chemical modification process. As Figure S2B displayed, the characteristic peak of GO appeared at 1350 cm^−1^ and 1600 cm^−1^ relating to D band and G band, respectively. The I_D_/I_G_ ratio of GO was 0.99, which is the consistence with pure GO, while the value of γ-CD-GO was 1.06, which is higher than GO and agrees well with the functionalized GO [51,52]. This result is caused by chemical functionalization of γ-CD process, increased the defect states of GO and weaken the graphitic nature. Data from X-ray photoelectron spectroscopy (XPS) measurement also verified chemical derivatization of γ-CD functionating routine (Figure S3A). It is obvious that a significant N1s peak enhanced in the resultant γ-CD-GO, suggesting a new amide bond turned out. Furthermore, the C1s spectra of could be deconvoluted into characteristic peaks of functional groups in GO (Figure S3C,D). The -COOH (291.1 eV) peak in GO is absent, and a new -CO-NH- (288.5 eV) peak appears in γ-CD-GO, which supported the chemical conjugation process. Moreover, as elemental analysis illustrates (Figure S3B), the growth in nitrogen content from 2.26% (GO) to 2.64% (γ-CD-GO), proving that the -NH_2_ groups in γ-CD have been successfully attached to GO. Additionally, the amount of γ-CD molecule decorating in GO was 35.70 wt% by estimation, nearly one γ-CD per 208 carbon atoms. In conclusion, all the above results indicated the γ-CD-GO biosensor were successfully assembled.

**Figure 1:**
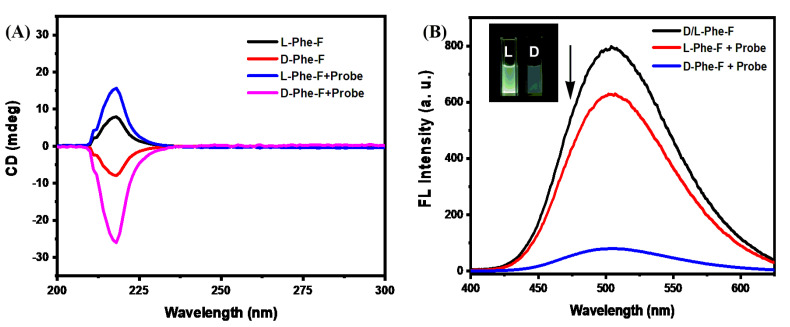
(**A**) Circular dichroism spectra of F-D/L-Phe (10^−3^ M) treated with same concentration of γ-CD-GO (0.05 mg/mL) dispersion; (**B**) Fluorescence assay for F-L/D-Phe (5 × 10^−6^ M), F-L-Phe with γ-CD-GO, and F D-Phe with γ-CD-GO in water; inset image: the fluorescence change of F D/L-Phe at the same concentrations of γ-CD-GO. The different variation of CD spectra and fluorescence response of γ-CD-GO against F-L/D-Phe clearly demonstrates the outstanding chiral sensing capacity of γ-CD-GO.

The chiral sensing ability of γ-CD-GO probe was first proven by circular dichroism spectroscopy (CD). As described in CD spectra from Figure 1A, the symmetrical peaks of F-D/L-Phe enantiomers at 217 nm were presented, which displaying the inherently chiral properties of F-D/L-Phe. While addition of γ-CD-GO (0.05 mg/mL) at same concentration to the aqueous solution of F-D/L-Phe (10^−3^ M), it is clear that γ-CD-GO exhibit different CD response signals towards F-D/L-Phe. The CD spectroscopy signal of F-D-Phe was sharply increased, while F-L-Phe showed a little CD change, indicating that the γ-CD-GO possessed prominent chiral sensing capacity for F-D-Phe enantiomers.

The chiral selectivity of γ-CD-GO against F-D/L-Phe enantiomers were further demonstrated by fluorometric studies. It is clear from Figure 1B that after the addition of γ-CD-GO into F-D/L-Phe solution, γ-CD-GO revealed a high selectivity of fluorescence quenching for F-D-Phe. In contrast, the fluorescence intensity of F-D/L-Phe was slightly enhanced by native γ-CD molecules according to the previous literature [53,54]. Because the fluorescence intensity of the dansyl moiety is sensitive to a micro-environment, which became stronger in a hydrophobic environment than in a hydrophilic environment towards host–guest interaction, leaded to a fluorescence enhancement of F-D/L-Phe. Taking the fluorescence enhancement of F-D/L-Phe by native γ-CD molecule into consideration, it is reasonably concluded that the fluorescence quenching of F-D/L-Phe was not ascribed to the energy transfer between the F-D/L-Phe and the mono-γ-CD, but the guest-inclusion interaction between F-D/L-Phe and γ-CD on the GO surface caused F-D/L-Phe approach to GO and induced the electron transfer from F-D/L-Phe to GO. As shown in inset photograph of Figure 1B, the fluorescence quenching was found to be enantioselective. After the addition of the γ-CD-GO of the same concentration, the luminescence intensity of F-D-Phe and F-L-Phe were quenched about 89.81% and 10.73% from the original value, respectively. The fluorescence decrease ratio for F-D-Phe is 8.36 times to F-L-Phe induced by γ-CD-GO, which demonstrates the excellent chiral selectivity of γ-CD-GO against the F-D/L-Phe enantiomers.

**Figure 2:**
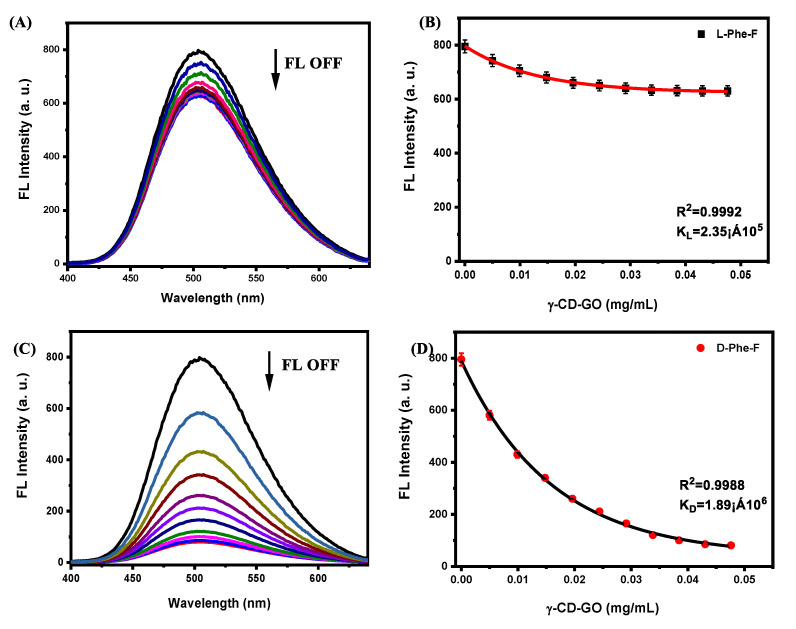
Fluorescence spectrum of (**A**) F-L-Phe, (**B**) F-D-Phe (5 × 10^−6^ M) incubated with a series of volumes (10–100 μL) of γ-CD-GO (0.05 mg/mL). Titration curves between γ-CD-GO and F-L-Phe (**C**), and F-D-Phe (**D**), respectively. The changes of fluorescence measurement obviously demonstrate the strong stereoselectivity of γ-CD-GO towards F-D/L-Phe.

**Figure 3:**
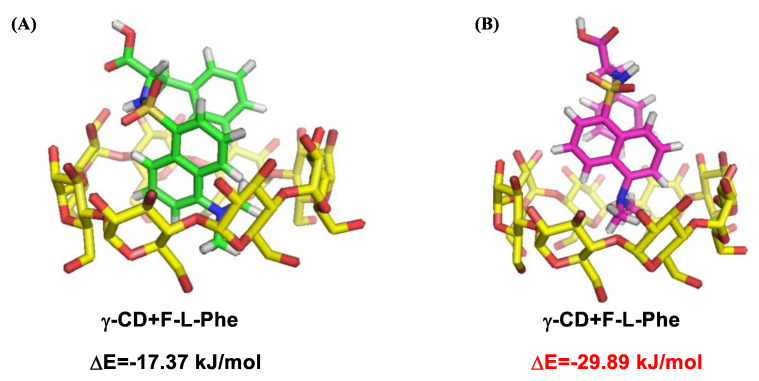
The complex modes formed by (**A**) γ-CD and F-L-Phe, (**B**) γ-CD and F-D-Phe were calculated at b3lyp/6−31G(d) levels using Gaussian 03 [1] package. The molecular calculation demonstrates strong stereoselectivity of γ-CD-GO towards F-D-Phe.

Subsequently, for quantifying the enantioselective chemical sensing properties of the γ-CD-GO, a fluorescence titration experiment was performed. Figure 2A,C depict the fluorescence spectrum of a series of γ-CD-GO treated with F-L-Phe and F-D-Phe, respectively. As the γ-CD-GO concentration increased, the fluorescence intensity reduction with F-D/L-Phe behaves enantio-differentiating. According to a nonlinear manner [29], the dependence of I as a function of c (c is the concentration of γ-CD-GO, and I is the fluorescence intensity of F-D/L-Phe at the given γ-CD-GO concentration) is plotted in Figure 2B,D. The binding constants B between γ-CD-GO and F-D-Phe were found to be 1.89 × 10^6^, while the binding constants B for F-L-Phe was 2.3 × 10^5^. It was found that the chiral discrimination ratio of K_D_/K_L_ = 8.21 for γ-CD-GO, which is 5.6 times higher than the native γ-CD (K_D_/K_L_ = 1.46) [20], manifesting this γ-CD-GO sensor exhibited an outstanding enantioselectivity for F-D-Phe in solution. The previous literature reported that the enantiomeric discrimination in favor of F-D-Phe is caused by the mode of deep guest penetration into the cavities of the γ-CD molecules [42]. Additionally, molecular simulation was performed at the b3lyp/6–31G (d) levels to evaluate the binding ability of probes to F-D/L-Phe. As Figure 3 shown, the binding energy of probe and F-L/D-Phe complex is −17.37 kJ/mol and −29.89 kJ/mol, respectively, which also verified the deep guest penetration and higher binding ability of probe to D-enantiomer. The detection limit for L- and F-D-Phe, estimated to be 52 × 10^−6^ M and 2.3 × 10^−6^ M based on the response of three times the blank solution, which is more sensitive compared to other approaches for quantification [29]. As a result, the fluorescence measurement and molecular calculation results revealed the strong stereoselectivity of γ-CD-GO towards F-D-Phe.

**Figure 4:**
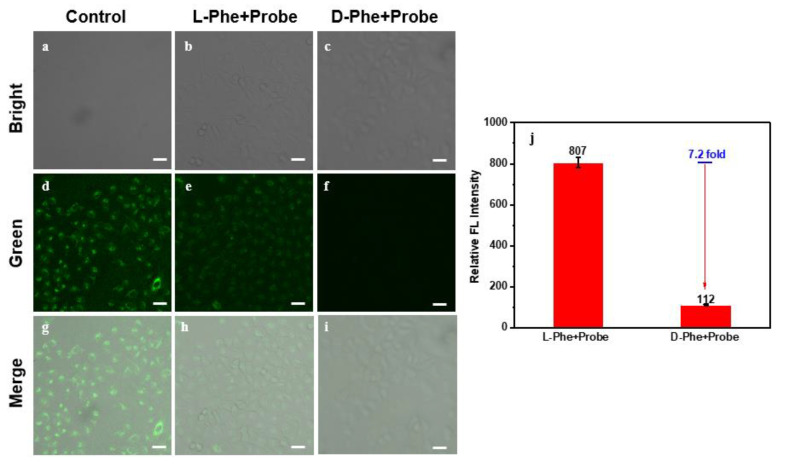
Chiral fluorescence labeling ability of γ-CD-GO in living cells. HeLa cells treated with L/D-Phe-F (**a**,**d**,**g**), L-Phe-F with γ-CD-GO (**b,e,h**), and D-Phe-F with γ-CD-GO (**c**,**f**,**i**). (**j**) Relative fluorescence intensity of representative images. The scale bar is 20 μm. The cell imaging assay indicates the remarkable chiral labelling ability of γ-CD-GO in living cells.

Due to the outstanding enantioselective sensing ability for this γ-CD-GO system, we then investigate its function in Hela cell line. To this end, the classical thiazolyl blue tetrazolium bromide (MTT) assays were completed to evaluate cell toxicity of γ-CD-GO. After 24 h incubation, the γ-CD-GO coexisted well with Hela cells (Figure S4). Therefore, Hela cells were first incubated with F-D/L-Phe, followed by γ-CD-GO probe treatment. As seen from the control in Figure 4d, after F-D/L-Phe incubation with the cell growth media for 2 h, bright green fluorescence was observed in intracellular, clearly illustrating that the F-D/L-Phe were cellular uptaken by Hela cells. Nevertheless, weaker fluorescence with obvious chiral selectivity for cells after the γ-CD-GO at the same concentration incubation for 2 h (Figure 4h,i). According to the previous literature [55,56], graphene-based materials could efficiently be transported into living cells and directly penetration of cell membranes. It is reasonably concluded that the cellular endocytosis with γ-CD-GO, resulting in the fluorescence quenching intracellular (Figure 4h,i). It is 7.2-fold brighter fluorescence signal in cells for F-L-Phe than F-D-Phe incubation (Figure 4j), demonstrating the preferable enantioselective probing capability of the γ-CD-GO inside the cells. More important, as shown in Figure S5, after the treatment with D/L-Phe-F for 2 h and a further incubation with a series of different concentrations for γ-CD-GO (0–0.1 mg/mL) for 2 h, the intracellular fluorescence was also chiral selectivity declined in favor of D-Phe-F. These results suggested that the remarkable chiral fluorescence imaging ability of γ-CD-GO in living cells.

**Figure 5:**
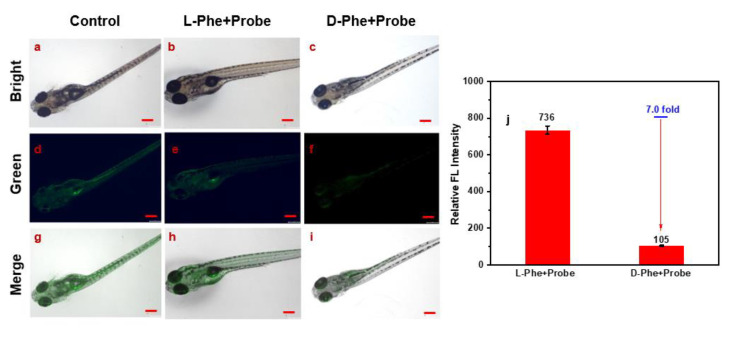
Fluorescence imaging of zebrafish incubated with F-L/D-Phe (**a**,**d**,**g**), F-L-Phe with γ-CD-GO (**b**,**e**,**h**), and F-D-Phe with γ-CD-GO (**c**,**f**,**i**). (**j**) Relative fluorescence intensity of representative images. The scale bar is 20 μm. The zebrafish imaging experiment indicated the remarkable chiral labelling ability of γ-CD-GO in model animals.

The sensing ability of γ-CD-GO to D-Phe in model animals is a vital factor to an ideal chiral probe. For biological applications, this highly sensitive chiral recognition system was also applied to more complex zebrafishes model animals. Zebra fish, first incubated in a solution of F-L/D-Phe, then treated with γ-CD-GO, which were observed under the fluorescence microscopy. As Figure 5g shown, the zebra fish exhibited a bright green fluorescence with incubation of F-L/D-Phe. After the zebrafish were treated with a γ-CD-GO solution with same concentration, the fluorescence was greatly decreased for F-D-Phe incubation in zebrafish (Figure 5i), while a slight fluorescence change for L-Phe-F incubation in zebrafish (Figure 5h). It is 7.0-fold brighter in fluorescence signal of the Zebrafish of the L-Phe-F treatment than those with D-Phe-F treatment (Figure 5j), distinctly visualizing the good chiral imaging property of γ-CD-GO in model animals. Moreover, seen from Figure S6, zebra fish with L-Phe-F incubation treated with different concentrations of γ-CD-GO (0–0.1 mg/mL) all show brighter fluorescence in contrast to zebrafish with D-Phe-F incubation. The zebrafish imaging experiment also displayed the γ-CD-GO bio-sensor could perform chiral imaging in living organisms with high chiro-selectivity and sensitivity.

## 3. Materials and Methods

### 3.1. Materials

All chemicals were AR grade and were purified by standard procedures. 1-Dimethylaminonaphthalene-5-sulphonyl chloride (dansyl chloride) and mono-6-amino-γ-cyclodextrin were purchased from Sigma-Aldrich. L/D-Phenylalanine, graphite, (3-dimethylaminopropyl)ethyl-carbodiimidmonohydrochloride (EDC), and N- hydroxysulfosuccinimide (sulfo-NHs) were purchased from Sinopharm Chemical Reagent Beijing Co., Ltd. (SCRC, Shanghai, China). All solutions were prepared in MilliQ water (18.2 MΩ).

### 3.2. Apparatus

The FT-IR spectra were recorded by a Thermo Nicolet NEXUS IR spectrometer with KBr disks. Fluorescence spectra were recorded on a FluoroMax-P luminescence spectrometer (HORIBA JOBIN YVON INC). The static contact angle was measured with a contact angle system (OCA 20, Dataphysics), and all of the photographs were taken by a digital camera. Circular dichroism (CD) spectra were measured on a JASCO J-810 spectropolarimeter at room temperature. The optical chamber of the CD spectrometer was deoxygenated with dry purified nitrogen (99.99%) for 45 min before use and the nitrogen atmosphere was retained during the experiments. The transmission electron microscopy images of graphene were recorded by a Philips TecnaiG2 TEM using an accelerating voltage of 200 kV. Tapping mode atomic force microscopy (AFM) characterization was conducted on a Nanoscope III (Digital Instrument) scanning probe microscope. X-ray photoelectron spectroscopy (XPS) images were recorded by a PHI Quantera SXM. Confocal images were acquired using a Zeiss confocal laser scanning unit mounted on an LSM710 fixed-stage upright microscope. Images were recorded using a 40× objective. The laser excitation for D/L-Phe-F is 390 nm, and the fluorescence detection band was set to 446–607 nm. The ultrasonic bath was an SB120D supersonic instrument.

### 3.3. Preparation of γ-CD-GO

GO was prepared from the graphite powder according to the Hummers method. A 10.0 mL portion of the GO dispersion (0.05 mg/mL) was mixed with 200 μL 2 mg/mL EDC and 50 μL 1 mg/mL NHs for 12 h at room temperature, followed by the addition of 20 mg NH_2_-γ-CD for overnight reaction. Finally, functionalized γ-CD-GO was treated by centrifugation and washed with water three times to remove the excess NH_2_-γ-CD. The final product was microscopically characterized by instruments.

### 3.4. Preparation of F-D/L-Phe

D- or L-phenylalanine dansyl chloride fluorescent derivatives (F-D/L-Phe) were synthesized via dansylation reaction according to the literature. [11] Briefly, 10 mL of D/L-phenylalanine aqueous solution (10^−2^ M) was mixed with 30 mL of bicarbonate solution (pH 10.5) in a stoppered glass tube, followed by the addition of 10 mL of freshly prepared dansyl chloride (1.5 × 10^−2^ M in acetonitrile) solution, the mixture was left standing in the dark for 30 min at room temperature. Then, the amino acids derivative was extracted dichloromethane on a vortex mixer three times, and the organic layer was separated and dried over anhydrous sodium sulfate. Finally, the resulting solution was dried in vacuum drier and diluted to make a F-D/L-Phe aqueous dispersion.

### 3.5. Cell Culture and Fluorescence Microscopy

Hela cells were grown in dulbecco’s modified eagles medium (DMEM, Invitrogen, USA) supplemented with 10% fetal calf serum (FBS). The cells were seeded in tissue culture plates and incubated in a fully humidified atmosphere at 37 °C containing 5% CO_2_. [29] Hela cells were grown on glass coverslips placed at the bottom of 6-well culture plates. In this experiment, the medium was replaced with fresh medium containing the materials F-D/L-Phe (10^−3^ M). After 2 h of treatment, the cells were washed three times with phosphate-buffered saline (PBS), and fluorescence images were taken with a confocal fluorescence microscope (Figure 4a,d,g). After D/L-Phe-F treating, Hela cells were incubated with fresh medium containing γ-CD-GO at a concentration of 0.1 μg/mL for another 2 h. Finally, the aforementioned cell was rinsed with PBS, then observed under a confocal fluorescence microscope (Figure 4b,c,e–i,h).

### 3.6. Chiral Imaging in Zebrafish

The 3 to 7 days postfertilization zebrafish were purchased from Eze-Rinka Company (Nanjing, China). The zebrafish were cultured in 5 mL of embryo medium supplemented with 1-phenyl-2-thiourea (PTU) in 6-well plates for 24 h at 30 °C. The zebrafish were first pre-treated with D/L-Phe-F (10^−3^ M) for 2 h, then the zebrafish were washed three times to remove D/L-Phe-F, the fluorescence images were taken with a confocal fluorescence microscope (Figure 5a,d,g). After that, the zebrafish were further incubated with γ-CD-GO (0.1 μg/mL) for 2 h at 30 °C. After removing the medium and washing zebrafish with PBS for three times, the fluorescence images were acquired with stereo microscopy (Figure 5b,c,e–i,h) (Olympus SZX16, Tokyo, Japan).

### 3.7. Data Analysis and Fitting

All fittings were performed in a nonlinear manner according to spectrofluorometric titrations [9]. For the direct host-guest titrations, the complexation process of the γ-CD-GO host (H) with the dye guest F-D/L-Phe (G) was expressed by Equation (1) according to a 1:1 host-guest binding stoichiometry, and the complex stability constant (Ka) is given by Equation (2).

(1) $$H+G\overset{K_{a}}{⇌}H\cdot G$$

(2) $$K_{a}=\frac{\left[H\cdot G\right]}{\left[H]\cdot [G\right]}$$

where ΔF and Δε′ denote the changes in the fluorescence intensity and molar extinction coefficient of the chromophoric β-CD-GO derivative upon inclusion complexation of the D/L-Phe-F, respectively.

ΔF = Δε′ [H·G] (3)

Solving the simultaneous equations, we obtained Equation (4).

(4) $$\Delta F=\frac{\Delta \varepsilon \prime ({\left[H\right]}_{0}{+[G]}_{0}+\frac{1}{K_{a}}\;)\pm \sqrt{\Delta \varepsilon^{\prime 2}{{\left([H\right]}_{0}+{\left[G\right]}_{0}+\frac{1}{K_{a}})}^{2}-4\Delta \varepsilon^{\prime 2}{\left[H\right]}_{0}{\left[G\right]}_{0}}}{2}$$

The complex stability constant Ka and the sensitivity factor Δε′ were calculated by nonlinear fitting using the value of ΔF observed at each initial guest concentration [G]_0_.

## 4. Conclusions

In summary, we propose a sample and convenient approach to fabricate chiral imaging sensor based on γ-CD-GO in zebrafish model animal. This chiral probe shows a high chiro-selectivity and sensitivity for D-Phe both in living cells and in zebrafish, and the chiral discrimination rate is K_L_/K_D_ = 8.21, which displays an excellent fluorescent chiral sensing probe in practical complex biological samples. It is anticipated that this study will make an important contribution to better understanding of D-Phe activity in living organisms, paving the way for the construction of practical chiral imaging devices.

## Data Availability

The data are available upon request to the authors.

## Supplementary Materials

The following supporting information can be downloaded at: https://www.mdpi.com/article/10.3390/molecules28093700/s1, Figure S1: TEM images of GO; Figure S2: (A) FT-IR spectra of pure γ-CD, GO, and γ-CD-GO, respectively; (B) The amplification of FT-IR spectra; (C) Raman spectra showing the D and G bands of GO and γ-CD-GO, respectively; Figure S3: (A) XPS spectrum of GO and γ-CD-GO; (B) Element content analysis of GO and γ-CD-GO; XPS C1s spectrum of (C) GO; (D) γ-CD-GO, respectively; Figure S4: Relative cell viability of Hela treated with γ-CD-GO in different concentrations for 24 h in fresh medium.; Figure S5: Confocal fluorescence microscopy images of Hela cells incubated with F-D/L-Phe (control L and D); Figure S6: Fuorescence microscopy images of zebra fish incubated with F-D/L-Phe (control L and D); Table S1: Thermodynamic parameters for the interaction of γ-CD with D/L-Phe-F by molecular simulation calculated at b3lyp/6-31G(d) levels using the Gaussian 03 [1] package.
