# Supplementary material for: Enantioselective Labeling of Zebrafish for D-Phenylalanine Based on Graphene-Based Nanoplatform
Source: Molecules. 2023 Apr 25;28(9):3700. doi: 10.3390/molecules28093700 (PMC10180043; doi:10.3390/molecules28093700)
Supplement: Supplementary file 1 [file molecules-28-03700-s001.zip › molecules-2284676-supplementary.pdf]

*molecules*

MDPI

# Enantioselective Labeling of Zebrafish for D-Phenylalanine Based on Graphene-Based Nanoplatform

*Supporting Information*

**Yuqing He [1,†], Ziqi Ye [2,†], Fei Zhu [3,†], Tianxiang Qiu [1], Xiyan Dai [1], Yue Xie [1], Shibiao Zou [1], Qingjian Dong [4,*], Weiying Zhang [2], Junkai Ma [5] and Xiaowei Mao [1,*]**

1 Hubei Key Laboratory of Environmental and Health Effects of Persistent Toxic Substances, School of Environment and Health, Jianghan University, Wuhan 430014, China
2 Key Laboratory of Optoelectronic Chemical Materials and Devices of Ministry of Education, School of Life Science, Jianghan University, Wuhan 430014, China
3 Hubei Key Laboratory of Embryonic Stem Cell Research, School of Basic Medical Science, Hubei University of Medicine, Shiyan 442000, China
4 Department of Nuclear Medicine, Tongji Hospital, Tongji Medical College, Huazhong University of Science and Technology, Wuhan 430074, China
5 Hubei Key Laboratory of Wudang Local Chinese Medicine Research, Department of Chemistry, School of Pharmacy, Hubei University of Medicine, Shiyan 442000, China
* Correspondence: dongqingjian@hust.edu.cn (Q.D); maoxiaowei5167@126.com (X.M.)
† These authors contributed equally to this work.

## Contents

1. TEM characterization of GO……………………………………………………S9
2. The FT-IR and IR spectra of GO and γ-CD-GO………………………………S10
3. XPS spectrum and elemental analysis of GO and γ-CD-GO …………………S11
4. The Gaussian calculation data……………………………………………….....S12
5. Cytotoxic of γ-CD-GO……………………………………………………………S13
6. Chiral imaging of γ-CD-GO in Hela cells………………………………………S14
7. Chiral imaging of γ-CD-GO in zebra fish ……………………...……………. S15

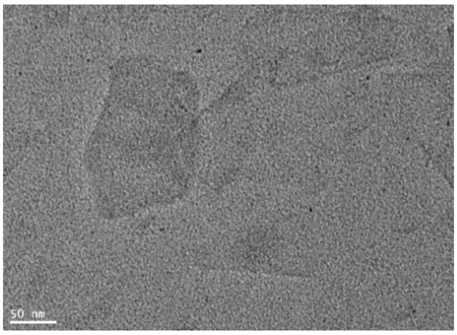


***Figure S1.*** TEM images of GO.

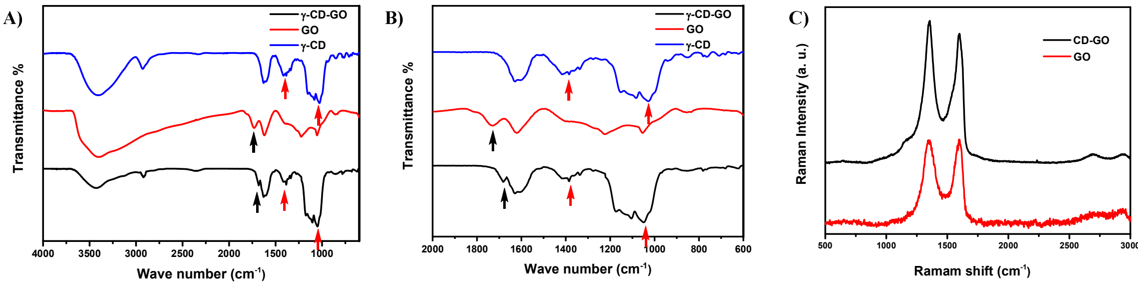


***Figure S2.*** A) FT-IR spectra of pure γ-CD, GO, and γ-CD-GO, respectively; B) The amplification of FT-IR spectra; C) Raman spectra showing the D and G bands of GO and γ-CD-GO, respectively.

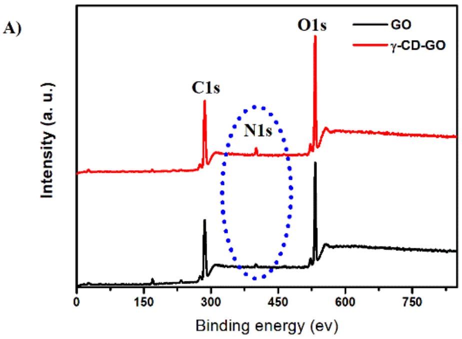


B)

| Element Assay | C | N | O |
| --- | --- | --- | --- |
| GO | 69.84 % | 2.26 % | 27.90 % |
| γ-CD-GO | 67.89 % | 2.86 % | 29.25 % |

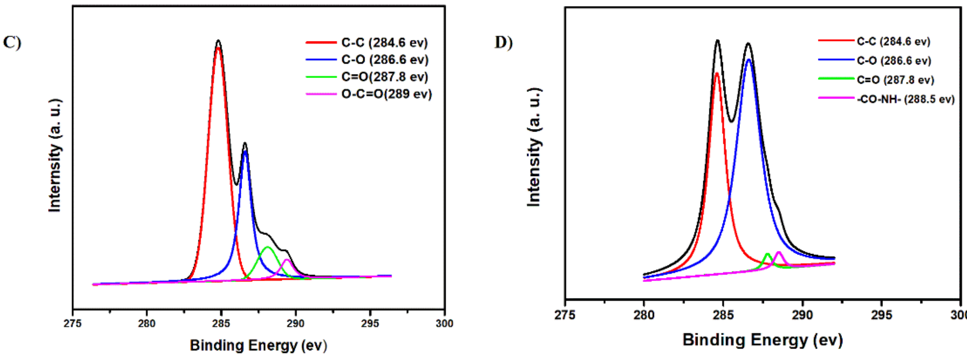


***Figure S3.*** A) XPS spectrum of GO and γ-CD-GO; B) Element content analysis of GO and γ-CD-GO; XPS C1s spectrum of C) GO; D) γ-CD-GO, respectively.

**Table S1.** Thermodynamic parameters for the interaction of γ-CD with D/L-Phe-F by molecular simulation calculated at b3lyp/6-31G(d) levels using the Gaussian 03[1] package.

| | Energy (a. u.) | ΔEnergy (a. u.) | ΔEnergy (kcal/mol) | ΔEnergy (kJ/mol) |
|---|---|---|---|---|
| γ-CD | -4858.55134324 | | | |
| F-D-Phe | -1613.83970198 | | | |
| F-L-Phe | -1613.84608168 | | | |
| F-D-Phe+γ-CD | -6472.40242796 | -0.01138274 | -7.14 | -29.89 |
| F-L-Phe+γ-CD | -6472.40402960 | -0.00660468 | -4.14 | -17.34 |

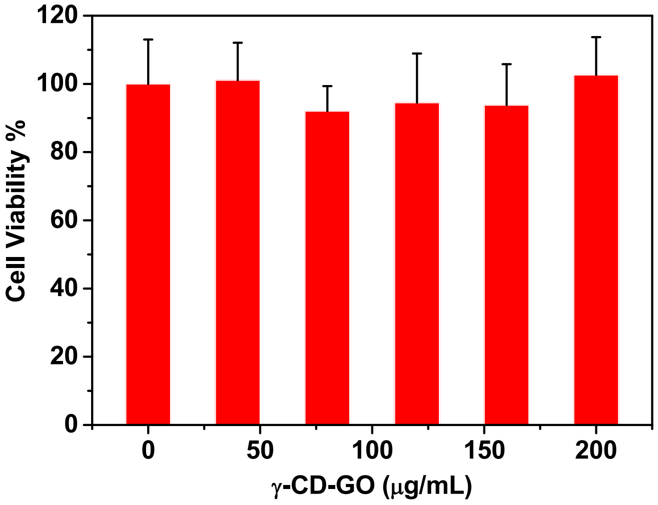


*Figure S4.* Relative cell viability of Hela treated with γ-CD-GO in different concentrations for 24 h in fresh medium.

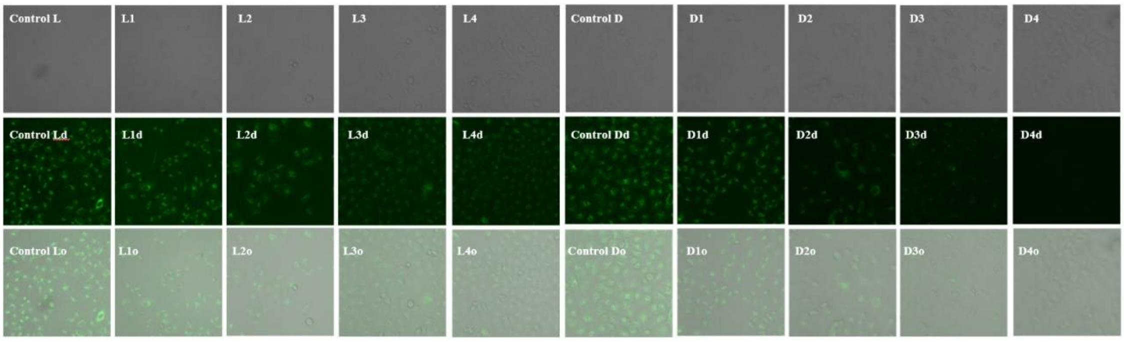


*Figure S5.* Confocal fluorescence microscopy images of Hela cells incubated with F-D/L-Phe (control L and D), or first incubated with F-D/L-Phe, then incubated with β-CD-GO in the concentration of 0.25 μg/mL (L1 and D1), 0.50 μg/mL (L2 and D2), 0.75 μg/mL (L3 and D3), and 1.00 μg/mL (L3 and D3) in sequence, and the images were obtained after extensive washing of cells with PBS for three times. Bright-field (top), dark-field fluorescence (middle), and overlap of images of dark and bright field (bottom), respectively.

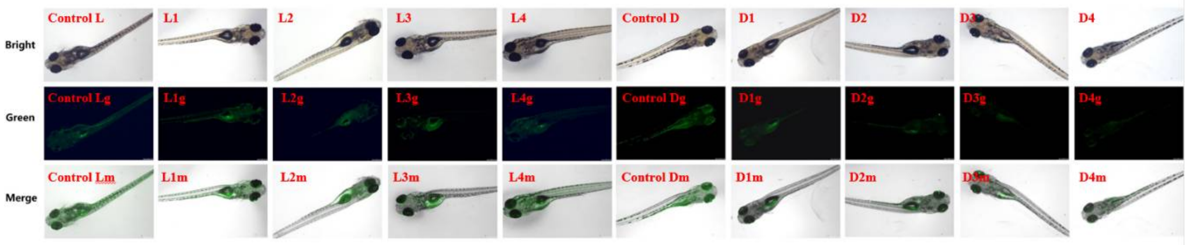


***Figure S6.*** Fuorescence microscopy images of zebra fish incubated with F-D/L-Phe (control L and D), or first incubated with F-D/L-Phe, then incubated with β-CD-GO in the concentration of 0.25 μg/mL (L1 and D1), 0.50 μg/mL (L2 and D2), 0.75 μg/mL (L3 and D3), and 1.00 μg/mL (L3 and D3) in sequence, and the images were obtained after extensive washing of cells with PBS for three times. Bright-field (top), dark-field fluorescence (middle), and overlap of images of dark and bright field (bottom), respectively.
